# Modeling scattered radiation from multi-leaf collimators (MLCs) to improve calculation accuracy of in-air output ratio

**DOI:** 10.1007/s13246-019-00781-2

**Published:** 2019-07-22

**Authors:** So-Yeon Park, Siyong Kim, Wonmo Sung, Sang-Tae Kim

**Affiliations:** 1 Institute of Radiation Medicine, Seoul National University Medical Research Center, Seoul, Republic of Korea; 2 Department of Radiation Oncology, Veterans Health Service Medical Center, Seoul, Republic of Korea; 3 Department of Radiation Oncology, Virginia Commonwealth University, Richmond, VA USA; 4 Department of Radiation Oncology, Massachusetts General Hospital and Harvard Medical School, Boston, MA USA; 5 Radiation Protection and Emergency Preparedness Bureau, Nuclear Safety and Security Commission, Seoul, Republic of Korea

**Keywords:** In-air output ratio, Intensity modulated radiation therapy, Volumetric modulated arc therapy, Source model, Multi-leaf collimator

## Abstract

This study aims to model an extra-focal source for the scattered radiation from multi-leaf collimators (MLCs), namely an *MLC scatter source*, and to correct in-air output ratio (*S*_*c*_) calculated using the conventional dual source model (DSM) to achieve better accuracy of point dose calculation. To develop the MLC scatter source, a 6 MV photon beam from a Varian Clinac® iX linear accelerator with millennium 120 MLCs was used. It was assumed that the position for the MLC scatter source was located at the center of the MLC, consisting of line-based and area-based sources to consider the characteristics of the scattered radiation from the MLCs empirically. Based on the measured *S*_*c*_ values for MLC-defined fields, optimal parameters for the line-based and area-based sources were calculated using optimization process. For evaluation of proposed method, measurements were taken for various MLC-defined square and irregular fields. The *S*_*c*_ values calculated using the proposed MLC scatter source and conventional DSM were compared with the measured data. For MLC-defined square fields, the measured *S*_c_ values showed better agreement with those calculated using the MLC scatter source (the mean difference was − 0.03%) compared with those calculated using the DSM (the mean difference was 0.18%). For MLC-defined irregular fields, the maximum dose differences between measurements and calculations using the MLC scatter source and DSM were 0.54% and 1.45%, respectively. The developed MLC scatter source could improve the accuracy of *S*_*c*_ calculation for both square and irregular fields defined by MLCs.

## Introduction

Following the advances in radiotherapy techniques, intensity modulated radiation therapy (IMRT) and volumetric modulated arc therapy (VMAT) have been widely adopted for various cancers, by delivering an optimal dose distribution to the target volumes while sparing the surrounding normal tissues. To ensure the required treatment accuracy of IMRT and VMAT, patient-specific quality assurance (QA) has become an important task in clinics. Generally, the calculated dose distributions are compared with the measured using several dosimeters for patient-specific QA. This process is labor intensive and time consuming. Hence, several institutions have tried to assure the deliverability of IMRT and VMAT plans using independent computer calculations instead of measurements [1–4]. In an independent verification program, the in-air output ratio (conventionally denoted as *S*_*c*_) for each beam segment must be accurately calculated to verify an IMRT or VMAT plan, which often comprises a significant number of beam segments or control points [2, 3].

There were three important factors to determine *S*_*c*_: (1) Scattered radiation from the linear accelerator head to the phantom (2) Backscattered radiation from the jaws to the monitor chamber, and (3) Source obscuring effect for small field sizes [5–9]. To calculate the *S*_*c*_ values, a source plane was introduced [10–13] and implemented through modeling methods such as single [14], dual [7], and three-source models [15]. Yan et al. compared the three models and demonstrated that the measured and calculated *S*_*c*_ values were in best agreement when using the dual-source model (DSM) [16]. In the DSM, a primary source at the position of the X-ray target and an extra-focal source at the position of the flattening filter were used. For an arbitrary field shape defined by both jaws and multi-leaf collimators (MLCs), the DSM was used to calculate *S*_*c*_ based on the detector’s eye view (DEV) and Gaussian integration methods [11].

The American Association of Physicists in Medicine (AAPM) Task Group (TG) 74 report clearly updated the definition of *S*_*c*_ as the change in the scattered radiation of the output beam due to the primary collimator, flattening filter, jaws, and MLCs [9]. Moreover, it stated that the scattered radiation from MLCs is no longer negligible for *S*_*c*_ calculation, particularly when small and irregular fields are used in IMRT and VMAT plans [9]. Nevertheless, the scattered radiation from MLCs has not been considered in previous source models, wherein MLCs only played a role of blocking the area to define the DEV. The DEV-based method cannot account for the effect of scattered radiation from MLCs when the leaves are not in the DEV field. Therefore, the calculated *S*_*c*_ of an MLC-defined field often differs from the measurement that originates from the MLC scatter effect. Several studies have been conducted to correct this discrepancy. Kim et al. quantified the amount of scattered radiation from the MLC of a Varian machine and attempted to include it in the *S*_*c*_ calculation using the equivalent field concept at the source plane and a field mapping method [11–13]. Alaei and Higgins introduced fitting curves for *S*_*c*_ to parameterize the aperture effect for any given jaw and MLC setting [17]. Zhu et al. developed an integrated extra-focal source involving MLC scatter and other components [18, 19]. Although these studies improved the calculation accuracy of *S*_*c*_ by accounting for the MLC scatter effect, residual discrepancies between the measured and calculated *S*_*c*_ values remained. The reason for these discrepancies was that scattered radiation from MLCs was considered as an independent source. To the best of our knowledge, no attempt has been made to explicitly model an individual extra-focal source accounting for only scattered radiation from MLCs.

This study aims to model an extra-focal source for the scattered radiation from MLCs, namely an *MLC scatter source*, and to correct *S*_*c*_ calculated using the conventional DSM to increase the accuracy of point dose calculation. The MLC scatter source was designed to have line-based and area-based sources for scattered radiation from the rounded-edge of MLCs and the exposed MLC areas, respectively, and then the parameters were iteratively optimized based on the measured *S*_*c*_. To validate the effectiveness of the proposed method, measurements were taken for various MLC-defined square and irregular fields. The *S*_*c*_ values calculated using the proposed MLC scatter source and conventional DSM were compared with the measured data.

## Methods

### Measurements for extra-focal source modeling

To model the extra-focal source, several sets of measured *S*_*c*_ values were obtained. A 6 MV photon beam from a Varian Clinac® iX linear accelerator equipped with 60 pairs of millennium MLCs (Varian Medical Systems, Palo Alto, CA, USA) was used. The measurements were taken using a 0.125 cm^3^ cylindrical ionization chamber (Model 31010, PTW-Freiburg, Germany) in a water-equivalent miniphantom (Model 670, CIRS Inc., Norfolk, VA, USA) at a depth of 10 cm. The source-to-chamber distance (SCD) was 100 cm.

For the conventional dual-source modeling, we measured *S*_*c*_ for various square and rectangular fields defined by the jaws with the MLCs fully retracted. On the other hand, to model the MLC scatter source, MLC-defined square fields were used with fixed jaw sizes ranging from 10 × 10 to 30 × 30 cm^2^. The MLC-defined square field sizes ranged from 4 × 4 cm^2^ to the fixed jaw-defined field size in increments of 1 cm. The reference field size was 10 × 10 cm^2^. In addition to the basic measurement data for modeling, the *S*_*c*_ values for various MLC-defined irregular fields were measured for evaluation. To reduce uncertainty in the *S*_*c*_ measurements, at least five readings were taken for every experiment and the standard deviations were within 0.2%. Table 1 summarizes the measurement cases.

**Table 1:** Measurement cases for modeling and evaluating the dual-source and multi-leaf collimator (MLC) scatter source

	Jaw-defined field (X × Y cm^2^)	MLC-defined field (X × Y cm^2^)
Dual-source model	4 × 4 to 40 × 40	Open (MLC retracted)
	10 × 4 to 10 × 40	
	4 × 10 to 40 × 10	
MLC scatter source model	10 × 10	4 × 4 to 10 × 10
	15 × 15	4 × 4 to 15 × 15
	20 × 20	4 × 4 to 20 × 20
	25 × 25	4 × 4 to 25 × 25
	30 × 30	4 × 4 to 30 × 30
For evaluation	15 × 15	Irregular shapes (cross, mirrored E, and maze)
	20 × 20	
	25 × 25	

*MLC* multi-leaf collimator

### Dual-source model (DSM)

The DSM proposed by Jiang et al. was chosen as the basic source model to which the MLC scatter source was added [7]. The DSM simultaneously accounts for the scattered radiation from the flattening filter and the backscattered radiation into the monitor chamber. *S*_*c,DSM*_ of a certain field size *fs* can be expressed as.

1 $$S_{c,DSM} (fs) = \frac{{\left( {1 + F_{efs} \left( {fs} \right)} \right) \cdot \left( {1 - F_{mbs} \left( {fs} \right)} \right)}}{{\left( {1 + F_{efs} \left( {fs_{ref} } \right)} \right) \cdot \left( {1 - F_{mbs} \left( {fs_{ref} } \right)} \right)}},$$

where *F*_efs_ is the ratio of the scattered radiation from the extra-focal source for flattening filter *ES*_sf_ to the contribution from the primary point source, *F*_mbs_ is the decreased ratio of the output beam due to the backscattered radiation into the monitor chamber (i.e., beam output ratio with the backscattered radiation to that without it), and *fs*_ref_ is the reference jaw setting (10 × 10 cm^2^ at the isocenter). The location of *ES*_sf_ is at the bottom of the flattening filter, and *F*_efs_ can be calculated by integrating over the back-projected area on *ES*_sf_ through the DEV [7, 10].

The DSM parameters in this study were iteratively optimized using the trust-region-reflective algorithm for nonlinear least squares (MathWorks, Inc., Natick, MA, USA) to match the calculated *S*_*c*_ with the measured one for the jaw-defined square and rectangular fields (see Table 1). The objective function was the chi-square difference between the measured and calculated *S*_c_ values for the jaw-defined fields with the MLCs fully retracted. The optimization was done within the maximum number of iterations (400) until the objective function was below a termination tolerance (10^−6^).

### MLC scatter source model

**Fig. 1:**
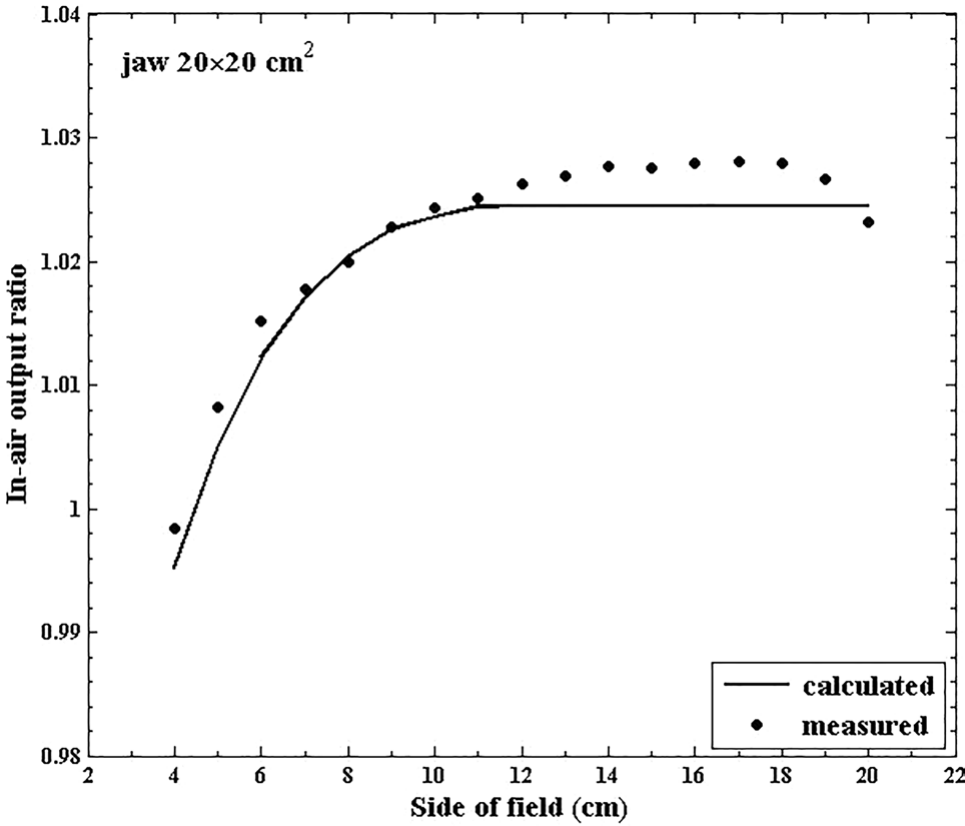
In-air output ratio (*S*_*c*_) for a 6 MV photon beam as a function of the multi-leaf collimator (MLC)-defined square field size ranging from 4 × 4 to 20 × 20 cm^2^ at a fixed jaw setting of 20 × 20 cm^2^. The calculated *S*_c_ values are derived from the dual-source model (DSM)

Basic concept of the MLC scatter source model: Fig. 1 shows a comparison of the measured *S*_*c*_ data with those calculated using the DSM for MLC-defined square field sizes ranging from 4 × 4 to 20 × 20 cm^2^ with a jaw setting of 20 × 20 cm^2^. As shown in Fig. 1, noticeable discrepancies are observed, implying that the scattered radiation from the MLCs influences *S*_*c*_. A correction factor was introduced to reduce the discrepancies as follows:

2 $$S_{c} = S_{c,DSM} \cdot S_{c,MLC} .$$

In Eq. (2), *S*_*c*,DSM_ is the DSM-calculated *S*_*c*_ value, and *S*_*c*,MLC_ is the correction factor for the scattered radiation from the MLCs. To determine *S*_*c*,MLC_ of an arbitrary field shaped by both jaws and MLCs, an MLC scatter source (*ES*_mlc_) was developed. It is assumed that the position for the *ES*_mlc_ is located at the center of the MLC (51.0 cm downward from the source target in this study). *ES*_mlc_ comprises line-based and area-based source models, namely *ES*_line_ and *ES*_area_, respectively, to consider the characteristics of the scattered radiation from the MLCs empirically.

3 $$S_{c,MLC} = S_{c,line} \cdot S_{c,area} .$$

*S*_*c*,MLC_ can be calculated by multiplying *S*_*c*,line_ and *S*_*c*,area_ using *ES*_line_ and *ES*_area_, respectively. *S*_*c*,line_ is a portion of the scattered radiation from the perimeter of the MLC-defined field (i.e., from the rounded-edge of MLCs). It increases when the MLC-defined field size increases or when it becomes irregularly shaped. *S*_*c*,area_ is a portion of the scattered radiation from the radiation-exposed area of the MLCs. It decreases when the MLC-defined field size increases and when the exposed MLC area decreases.

### Classification of the MLC scatter source model based on beam’s eye view (BEV) and DEV

**Fig. 2:**
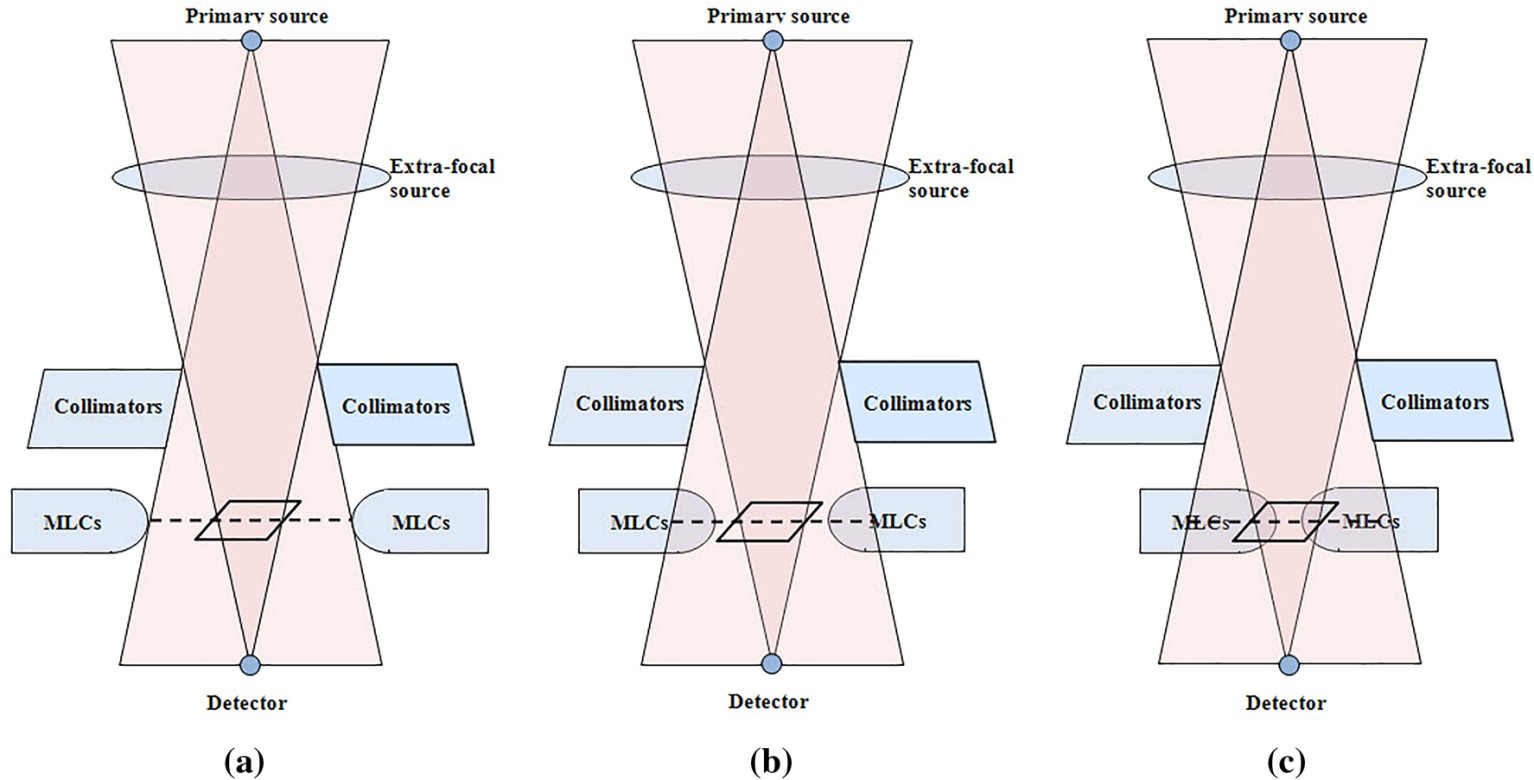
Schematics of the geometrical relationship between the jaws and multi-leaf collimators (MLCs) in terms of the beam’s eye view (BEV), detector’s eye view (DEV), and scatter interface for (**a**) category 1, (**b**) category 2, and (**c**) category 3

Based on the pattern of the discrepancy between the measured and calculated *S*_*c*_ values, as shown in Fig. 1, we designed *ES*_mlc_ to account for both BEV and DEV of a certain MLC-defined field. Three categories are established as shown in Fig. 2: (1) The MLC is in a retracted position out of the jaw-defined BEV (2) The MLC is in the jaw-defined BEV but does not affect the change in the DEV, and (3) The MLC is in the jaw-defined BEV and simultaneously affects the change in the DEV.

To distinguish the categories 2 and 3, a rectangular plane, i.e., a *scatter interface*, was introduced. As shown in Fig. 2, the scatter interface is defined as an area projected by the jaw-based DEV at the mid MLC plane. Details regarding the scatter interface are given in the next section.

In category 1, the effect of *ES*_mlc_ was negligible or non-existent (i.e., *S*_*c*,MLC_ = 1). On the other hand, two individual *ES*_mlc_ values were required to explain the categories 2 and 3, wherein the MLC scatter contributions were assumed to be different. Therefore, using the scatter interface as a border, *ES*_mlc_ was derived.

### Scatter interface

**Fig. 3:**
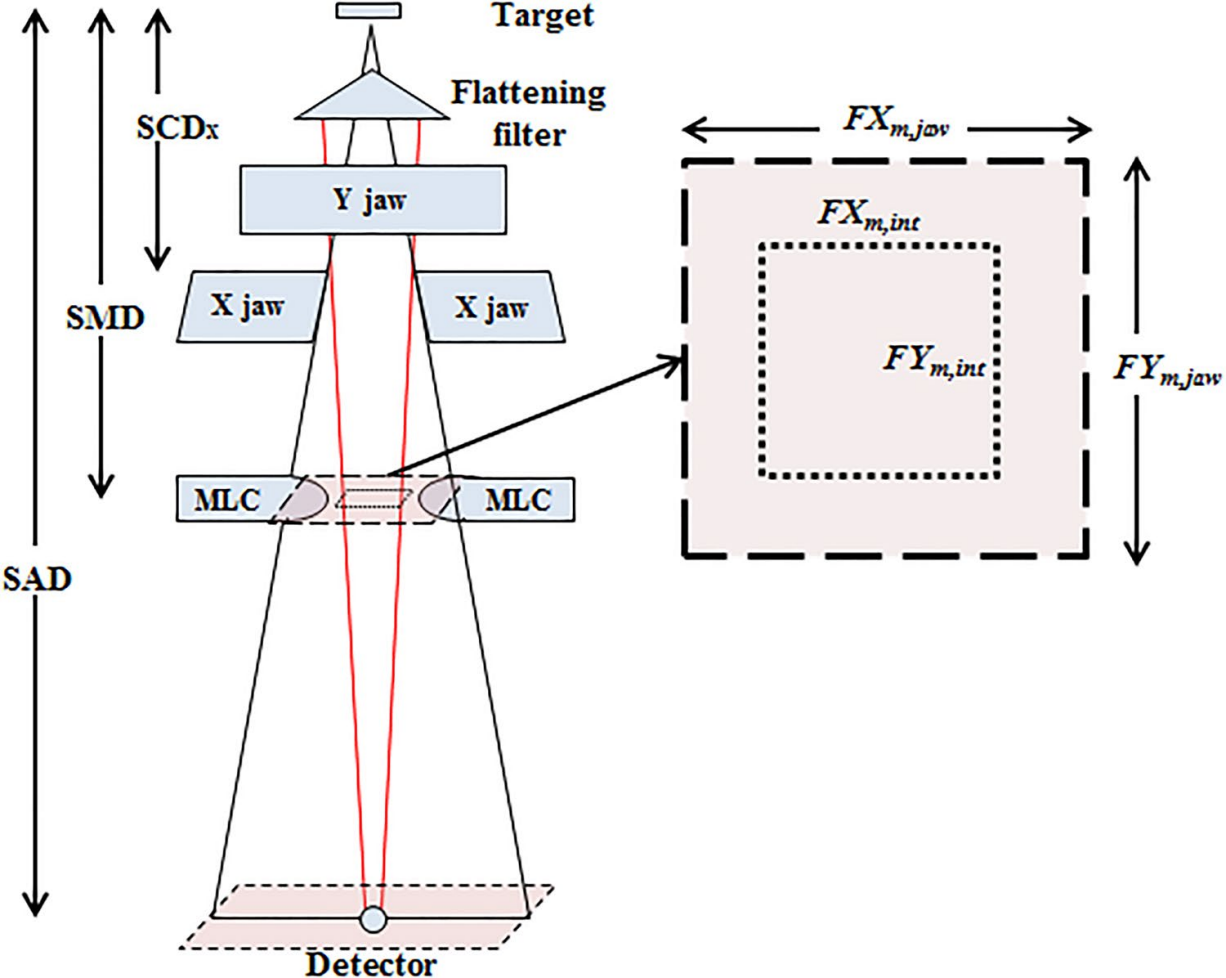
Schematic of the scatter interface geometry. With the constant *α*, the geometry of the scatter interface can be determined based on the jaw opening

Figure 3 shows a schematic of the geometry of the scatter interface in a linear accelerator. As mentioned above, the scatter interface size varies with respect to the jaw-defined field size. According to the geometrical relationship between the jaws and the scatter interface, a constant coefficient α was determined to calculate the scatter interface size as follows:

4 $$FX_{{m,{{int}} }} = \alpha \times FX_{m,jaw} ,$$

5 $$FY_{{m,{{int}} }} = \alpha \times FY_{m,jaw} ,$$

where *FX*_m,int_ and *FY*_m,int_ are the x and y sizes of the scatter interface at the mid-MLC plane, respectively, and *FX*_m,jaw_ and *FY*_m,jaw_ are the x and y sizes of the jaw-defined field at the same plane, respectively. From Fig. 3, α can be calculated as follows:

6 $$\alpha = \frac{{SCD_{x} \cdot \left( {SAD - SMD} \right)}}{{SMD \cdot \left( {SAD - SCD_{x} } \right)}}\left( {0.577\;{\text{in}}\;{\text{this}}\;{\text{study}}} \right),$$

where *SCD*_*x*_ is the distance between the source and the upper edge of the X jaw, *SMD* is the distance from the source to the mid MLC plane, and *SAD* is the source-to-axis distance.

### Empirical analysis of the MLC scatter source model

To account for the scatter contribution from the MLCs, *ES*_line_ and *ES*_area_ were assumed. *ES*_mlc_ can be described as follows:

7 $$ES_{mlc,out} \supset ES_{line,out}\, \& \,ES_{area,out} ,$$

8 $$ES_{mlc,in} \supset ES_{line,in}\, \& \,ES_{area,in} ,$$

where *ES*_line,out_ and *ES*_line,in_ denote the scattered radiations from the perimeter of the MLC-defined fields (reaching to the point of interest) in categories 2 and 3, respectively, as shown in Fig. 2. *S*_*c*,line_ can be directly calculated using the source models with the perimeters of the MLC-defined fields.

We chose a fitting function to express *ES*_line_ as follows:

9 $$ES_{line} = a\left( {FP^{b} - RP^{b} } \right) + 1,$$

where *FP* is the perimeter of the MLC-defined field. *RP* is the perimeter of the scatter interface. For categories 2 (i.e., outside the scatter interface) and 3 (i.e., inside the scatter interface), two parameters *a* and *b* need to be determined for the line-based source.

*ES*_area,out_ and *ES*_area,in_ denote the scattered radiations from the radiation-exposed area of the MLCs in categories 2 and 3, respectively. *ES*_area_ can be calculated as follows.

10 $$ES_{area} = e^{{ - MA/2\sigma^{2} }} .$$

In Eq. (10), σ is a parameter of the Gaussian distribution. *MA* is the exposed MLC area at the mid-MLC plane and was calculated by subtracting the MLC-defined field size from the jaw-defined field size. From empirical evidence, it was found that the optimal parameters which were *a*, *b*, and σ for the MLC scatter sources vary with different field sizes defined by the jaws and type of category. These parameters were empirically– determined as a quadratic function of the area to perimeter (AP) ratio of the jaw-defined field size as follows:

11 $$\begin{gathered} \left\{ {\begin{array}{*{20}l} {a_{out} = 3.067e^{ - 5} \cdot A\Pr atio^{2} + 5.333e^{ - 5} \cdot A\Pr atio + 0.015} \\ {b_{out} = 7.810e^{ - 4} \cdot A\Pr atio^{2} - 6.042e^{ - 3} \cdot A\Pr atio + 0.840} \\ {\sigma_{out} = - 1.762e^{ - 4} \cdot A\Pr atio^{2} + 26.360e^{ - 5} \cdot A\Pr atio - 37.103} \\ \end{array} } \right. \hfill \\ \left\{ {\begin{array}{*{20}l} {a_{in} = 3.724e^{ - 4} \cdot A\Pr atio^{2} + 5.120e^{ - 5} \cdot A\Pr atio + 0.025} \\ {b_{in} = 3.303e^{ - 4} \cdot A\Pr atio^{2} - 4.135e^{ - 3} \cdot A\Pr atio + 1.648} \\ {\sigma_{in} = - 14.072e^{ - 4} \cdot A\Pr atio^{2} + 224.207e^{ - 3} \cdot A\Pr atio - 440.218} \\ \end{array} } \right. \hfill \\ \end{gathered}$$

Similarly, the parameters were optimized using the same optimization methods as in DSM to attain the best fit between the measured and calculated *S*_*c*,MLC_ values.

### General formalism of S_c,MLC_ for an arbitrary field shape

**Fig. 4:**
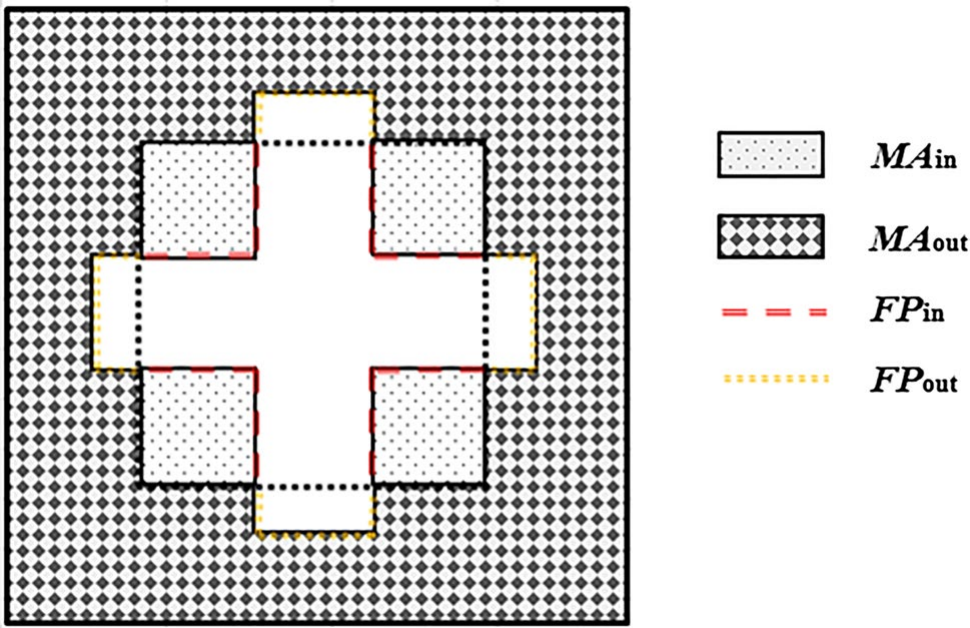
Schematic of *S*_*c*,MLC_ calculation for an arbitrary irregular multi-leaf collimator (MLC)-defined field using the MLC scatter source and the scatter interface. Here, *MA*_in_ and *MA*_out_ are the exposed MLC areas inside and outside the scatter interface, respectively. *FP*_in_ and *FP*_out_ are the perimeters of the MLC-defined field inside and outside the scatter interface, respectively

For an arbitrary irregular field defined by the MLCs, as shown in Fig. 4, *S*_*c*,MLC_ can be calculated by multiplying the contributions from the two regions divided by the scatter interface. A general formula for *S*_*c*,MLC_ is given as follows:

12 $$S_{c,MLC} = \left\{ {ES_{line,out} \left( {FP_{out} } \right) \cdot ES_{line,in} \left( {FP_{in} } \right)} \right\} \cdot \left\{ {ES_{area,out} \left( {MA_{out} - RA_{out} } \right) \cdot ES_{area,in} \left( {MA_{in} - RA_{in} } \right)} \right\},$$

where *MA*_in_ and *MA*_out_ are the radiation-exposed areas of the MLCs inside and outside the scatter interface, respectively. *FP*_in_ and *FP*_out_ are the perimeters of the MLC-defined field inside and outside the scatter interface, respectively. *RA* is a reference for the radiation-exposed areas of the MLCs (scatter interface area and 3 × 3 cm^2^ at the isocenter for categories 2 and 3, respectively).

### Evaluation of the MLC scatter source model

The calculated *S*_c_ values for various MLC-defined square and irregular fields were compared with the corresponding measurement values to evaluate the efficacy of the developed MLC scatter source. The size of the MLC-defined squares ranged from 4 × 4 cm^2^ up to the fixed position of the jaw-defined field sizes (ranging from 10 × 10 to 30 × 30 cm^2^). For the evaluation, the data sets with jaw-defined field sizes of 15 × 15 and 25 × 25 cm^2^ were added to the data sets previously used for modeling *ES*_mlc_. The *S*_c_ measurements were taken at least five times using a cylindrical ionization chamber at a depth of 10 cm (SSD: 90 cm) in a water-equivalent miniphantom and then averaged.

**Fig. 5:**
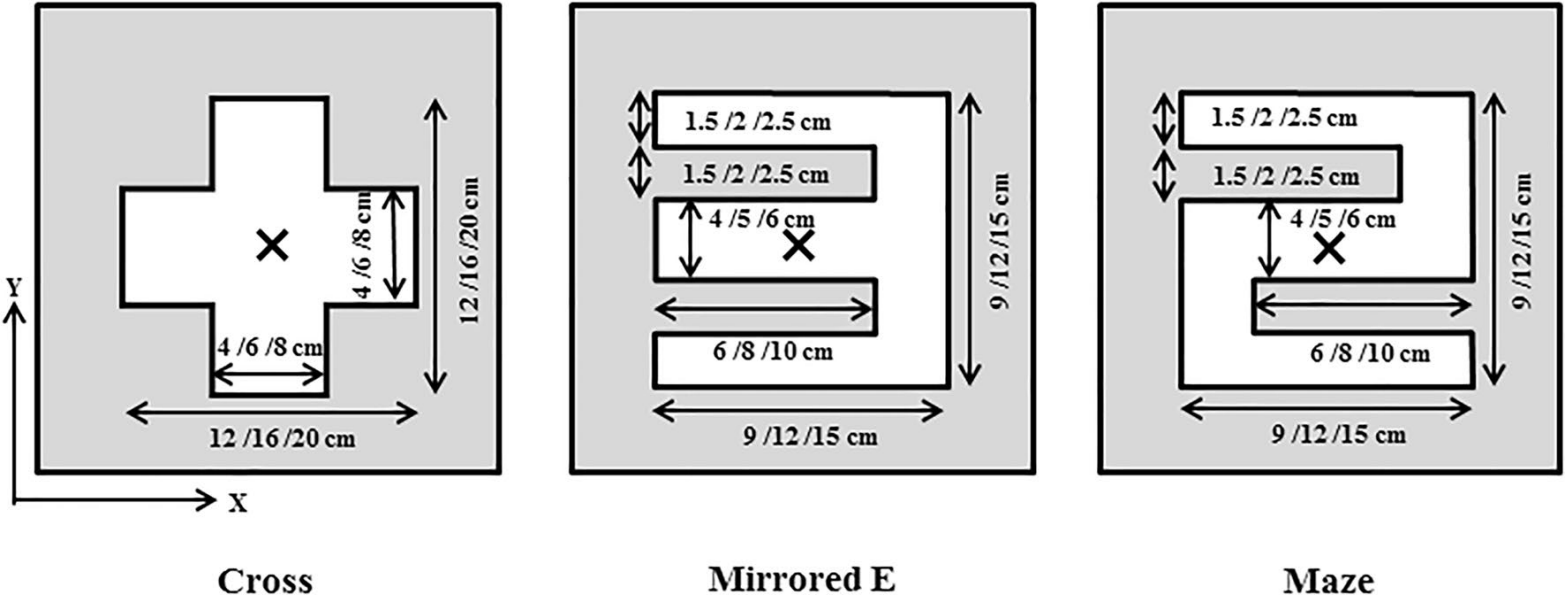
Three multi-leaf collimator (MLC)-defined irregular fields designed to verify the MLC scatter source. The irregular fields are used in three different sizes in accordance with jaw-defined field sizes of 15 × 15, 20 × 20, and 25 × 25 cm^2^

In addition, three irregular field shapes (cross, mirrored E, and maze) were considered, as shown in Fig. 5. Each irregular shape included three different sizes in accordance with jaw-defined field sizes of 15 × 15, 20 × 20, and 25 × 25 cm^2^ [20]. For the MLC-defined irregular fields, the dose and *S*_c_ values were calculated and measured at the central point. When measuring *S*_c_ values for irregular shaped fields, a brass build-up cap with a diameter of 1 cm was used as a miniphantom to provide electron equilibrium conditions. Clarkson’s method was used for the dose calculation, which integrates the scattered component of each angular sector for irregularly shaped fields [21]. With the Clarkson integration, values of phantom scatter factor (*S*_p_) and tissue maximum ratio (TMR) for three irregular field shapes were obtained by summing scatter contributions from each angular section of 1° (total angular section is 360°). Dose calculations with these parameters were performed by using an in-house program that was written in MATLAB (R2016a, Mathworks Inc., Natick, MA, USA). For the dose measurements, at least five readings were taken using a cylindrical ionization chamber at a depth of 10 cm (SSD: 90 cm) in a solid water phantom with a size of 30 × 30 × 30 cm^3^ and then multiplied by dose conversion factor (cGy/nC) to obtain the measured dose values. The dose conversion factors could be obtained by using measured readings for a given dose value.

## Results

### Dual-source model evaluation

**Fig. 6:**
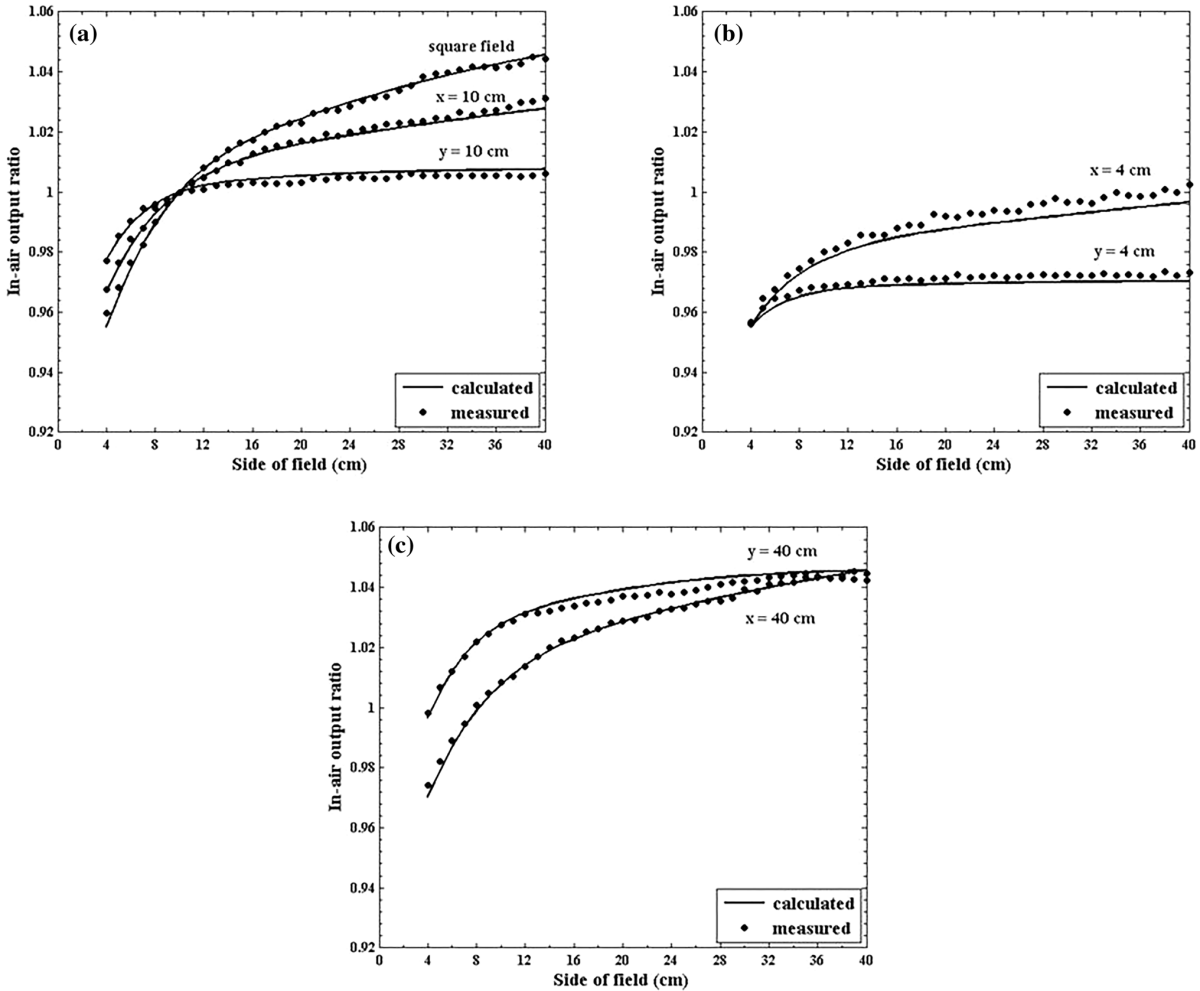
Comparison between the calculated and measured *S*_c_ values of a 6 MV photon beam from a Varian machine for (**a**) square fields ranging from 4 × 4 to 40 × 40 cm^2^ and rectangular fields with one pair of jaws fixed at 10 cm, (**b**) rectangular fields with one pair of jaws fixed at 4 cm while the other pair is varied from 4 to 40 cm, and (**c**) rectangular fields with one pair of jaws fixed at 40 cm while the other pair is varied from 4 to 40 cm. The conventional dual-source model is used for the calculation

The DSM in this study was evaluated using jaw-defined square fields ranging from 4 × 4 to 40 × 40 cm^2^ and rectangular fields with one pair of jaws fixed at 4, 10, or 40 cm while the other pair is varied from 4 to 40 cm. Figure 6 shows a comparison between the calculated and measured *S*_c_ values for seven sets of fields. The calculated and measured *S*_c_ values were in good agreement (the difference was lower than 0.47%) for square fields and rectangular fields with one pair of jaws fixed at 10 cm. The maximum discrepancies were 0.61% and 0.39% for the rectangular fields with one pair of jaws fixed at 4 and 40 cm, respectively. It was demonstrated that the DSM could accurately predict *S*_c_ for the jaw-defined fields with the MLCs fully retracted.

### MLC scatter source model evaluation

The optimum values of the parameters of *ES*_mlc,out_ and *ES*_mlc,in_ were determined through a nonlinear least squares method using the trust-region-reflective algorithm. Table 2 lists the results. The values of the line-based and area-based source models did not show a noticeable trend.

**Table 2:** Optimal parameter values of the multi-leaf collimator (MLC) scatter source for a 6 MV beam

Parameters	Jaw-defined field size X × Y (cm^2^)		
	10 × 10	20 × 20	30 × 30
*ES*_mlc,out_			
* a*_*out*_	0.016	0.016	0.017
* b*_*out*_	0.820	0.797	0.751
* σ*_*out*_	17.891	45.942	403.412
*ES*_mlc,in_			
* a*_*in*_	0.014	0.009	0.007
* b*_*in*_	0.503	0.456	0.404
* σ*_*in*_	32.418	57.543	450.124

Parameters were used in Eq. (12)

**Fig. 7:**
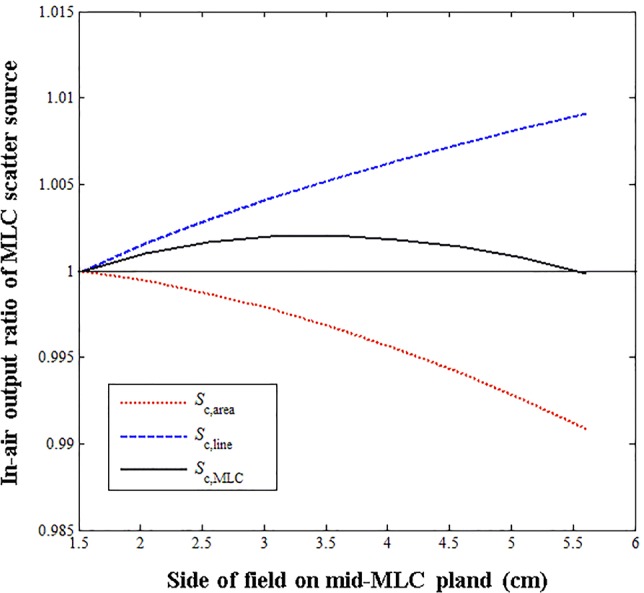
*S*_*c*,line_ and *S*_*c*,area_ calculated from multi-leaf collimator (MLC) scatter source comprising line-based and area-based source models as a function of the MLC-defined field at the mid-MLC plane. The calculated *S*_*c*,MLC_ values are determined by multiplying *S*_*c*,line_ and *S*_*c*,area_

The sources were empirically assumed based on the characteristics of the scattered radiation from the MLCs. Figure 7 shows the tendencies of the scattered radiation from the line-based and area-based sources. With the increase in the MLC-defined field size under the fixed jaw condition, the perimeter of the MLC-defined field increases, thereby increasing the scattered radiation from the perimeter. On the other hand, the scattered radiation from the radiation-exposed area of the MLCs decreases.

### Evaluation of MLC-defined square fields

**Fig. 8:**
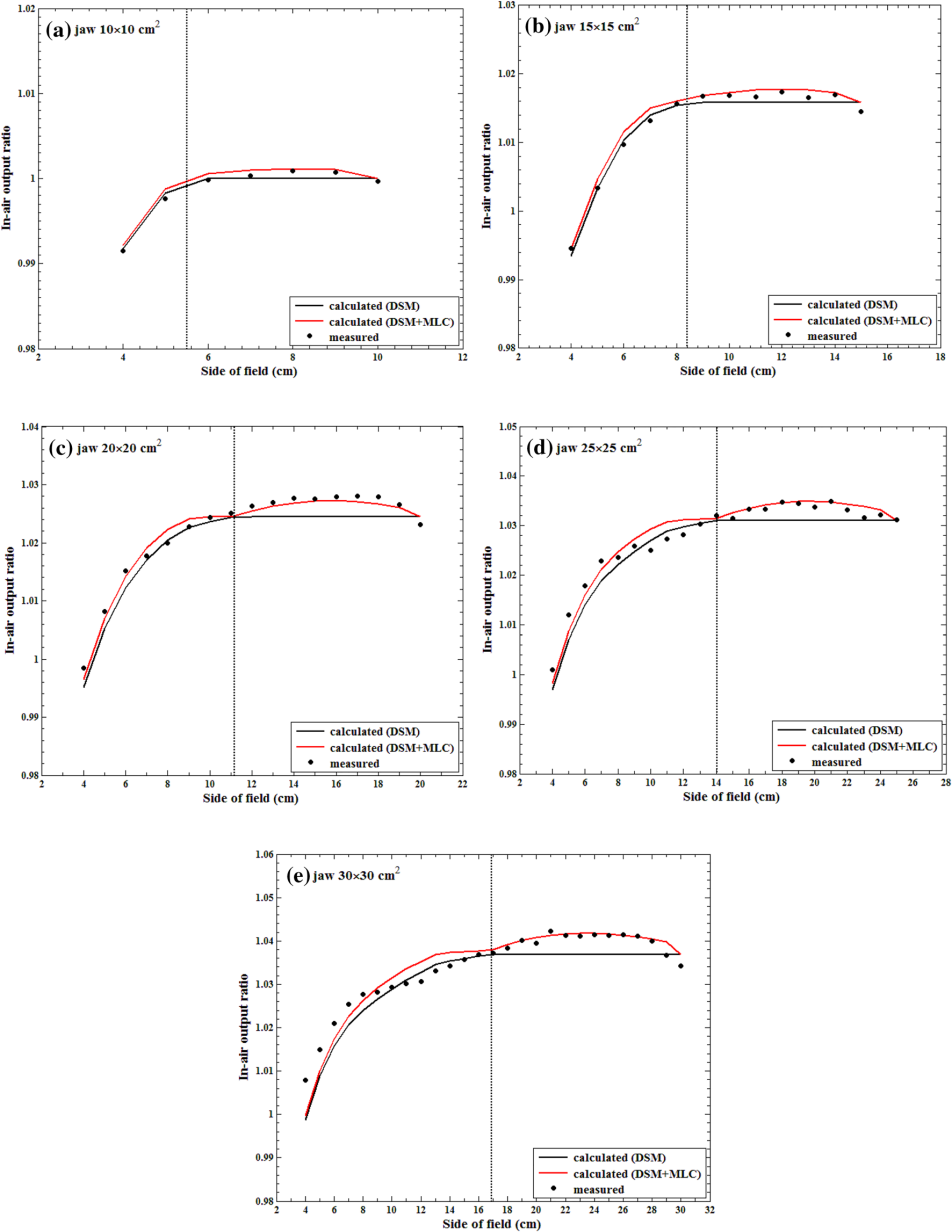
Comparison between the measured and calculated *S*_c_ values. *S*_c_ is calculated based on the dual-source model (DSM) and DSM in conjunction with the multi-leaf collimator (MLC) scatter source (DSM + MLC). The dashed line in each graph indicates the scatter interface for each jaw-defined square field

Figure 8 shows a comparison between three data sets: measured *S*_c_, *S*_c_ calculated using the DSM (i.e., DSM only), and *S*_c_ calculated using the DSM in conjunction with the MLC scatter source (denoted as DSM + MLC). The mean differences between the measured and calculated *S*_c_ values in the DSM case were 0.03%, 0.07%, 0.19%, 0.16%, and 0.25% for jaw-defined field sizes of 10 × 10, 15 × 15, 20 × 20, 25 × 25, and 30 × 30 cm^2^, respectively, whereas these values were − 0.06%, − 0.06%, − 0.03%, − 0.04%, and − 0.00% in the DSM + MLC case. The DSM + MLC results showed improved *S*_c_ calculation accuracy compared with the DSM results with statistical significance (*p* value < 0.03). When the MLC-defined fields were smaller than 8 × 8, 13 × 13, and 15 × 15 cm^2^ for jaw-defined field sizes of 20 × 20, 25 × 25, and 30 × 30 cm^2^, the difference between the DSM calculated and measured *S*_c_ values was less initially but increases thereafter. This is also due to only modeling the scattered radiation from linac components above the jaw in addition to interacting on the complicated contributions of the scattered radiation from the MLCs and jaws. Table 3 summarizes the numerical results of the differences between the measured and calculated *S*_c_ values for the MLC-defined square fields.

**Table 3:** Differences between the measured and calculated *S*_*c*_ values for multi-leaf collimator (MLC)-defined square field sizes ranging from 4 × 4 cm^2^ to the fixed jaw opening size

Jaw-defined field size (cm^2^)	DSM		DSM + MLC (this work)		*p* value
	Mean (%)	Maximum (%)	Mean (%)	Maximum (%)	
10 × 10	0.03	0.09	− 0.06	0.06	0.034
15 × 15	0.07	0.15	− 0.06	0.12	0.022
20 × 20	0.19	0.34	− 0.03	0.18	< 0.001
25 × 25	0.16	0.50	− 0.04	0.32	< 0.001
30 × 30	0.25	0.92	0.00	0.81	< 0.001

*DSM* dual-source model developed by Jiang et al., *DSM* + *MLC* dual-source model in conjunction with MLC scatter source developed in this study

### Evaluation of MLC-defined irregular fields

Table 4 lists the evaluation results of the MLC-defined irregular fields. The measured and calculated values of both *S*_c_ and dose were compared at the central point (shown in Fig. 5). For the cross field shape with three different sizes of 15 × 15, 20 × 20, and 25 × 25 cm^2^, the deviations between the measured and calculated *S*_c_ values in the DSM case were 0.64%, 0.52%, and 0.21%, respectively, whereas the values were 0.27%, − 0.15%, and − 0.11% in the case of DSM + MLC. For the mirrored E field shape with three different sizes of 15 × 15, 20 × 20, and 25 × 25 cm^2^, the deviations in the dose values in the DSM case were 1.45%, 0.79%, and 0.86%, respectively, whereas the values were 0.37%, 0.45%, and 0.25% in the DSM + MLC case. The larger the irregular field, the better the agreement between the measured and calculated values. Similar to the square field results, the irregular field results showed that the MLC scatter source could improve the *S*_c_ calculation accuracy and therefore the dose calculation accuracy with statistical significance (*p* value < 0.05).

**Table 4:** Comparison between the measured and calculated *S*_c_ values and dose values for irregular fields

Jaw-defined field size (cm^2^)	*S*c		Dose	
	Dev. (%) in DSM	Dev. (%) in DSM + MLC (this work)	Dev. (%) in DSM	Dev. (%) in DSM + MLC (this work)
Cross				
15 × 15	0.64	0.27	0.98	0.48
20 × 20	0.52	− 0.15	1.23	0.54
25 × 25	0.21	− 0.11	0.54	0.23
Mirrored E				
15 × 15	0.85	0.22	1.45	0.37
20 × 20	0.53	0.14	0.79	0.45
25 × 25	0.46	0.11	0.86	0.25
Maze				
15 × 15	0.25	0.15	0.34	− 0.02
20 × 20	0.21	0.10	0.41	0.03
25 × 25	0.63	− 0.02	0.91	0.12

*Dev* deviation, *DSM* dual-source model developed by Jiang et al., *DSM* + *MLC* dual-source model in conjunction with MLC scatter source developed in this study

## Discussion

This study attempted to explicitly model the scattered radiation from MLCs as an additional extra-focal source, which was not considered in conventional models including the DSM developed by Jiang et al. Jiang et al. reported that *S*_*c*_ calculated using the DSM through the DEV was lower than the measured value for certain MLC-defined fields [7]. This was because the model was intended only for jaw-defined fields, and the energy fluence variation was irregular depending on where the MLCs were located in terms of blocking the DEV, as shown in Fig. 1. To account for the irregularity, a simple Gaussian approximation as the extra-focal source may not be sufficient. Previous studies used a curve fitting method and an equivalent field method to calculate *S*_*c*_ for MLC-defined small and irregular fields [7, 11–13, 17]. On the other hand, Zhu et al. developed an algorithm based on an empirical model wherein the MLC scatter contribution was implicitly included [19]. The effective head scatter sources for the flattening filter and MLCs could be simultaneously determined using a scaling factor. Compared with the source model proposed by Zhu et al*.*, our MLC scatter source was independently developed to explicitly account for the MLC scattered radiation, and the limitation in defining the DEV in the conventional DSM was overcome by adding the MLC scatter source [19].

Both the DSM developed by Jiang et al. and MLC scatter source model developed in this study are fundamentally based on the measured *S*_*c*_ values, and it was demonstrated that it should be ensured that these data have small uncertainty. During measurements, there were several uncertainties related to field apertures defined by jaw or MLCs, cylindrical ionization chamber position in miniphantom, and Linac output variation. In this study, to reduce these uncertainties, several measurements were taken to achieve a small standard deviation of 0.2%. All data were qualified to model the sources and verify them.

The scatter interface is the boundary of the jaw-based DEV located at the mid-MLC plane and can be easily calculated using the parameter α for a given field size. It is used to distinguish between categories 2 and 3 geometrically and to define the boundary of approximately 50% of the beam intensity due to primary and scattered radiations for a certain jaw-defined field shape at the mid-MLC plane physically. The spatial beam intensities on the mid-MLC plane were calculated using the DSM and then normalized using the central intensity. The beam intensity inside the scatter interface has higher photon energy than outside the scatter interface because it might be based on several Gaussian-distribution sources from the target and flattening filter, and peripheral scattered radiation generally consists of low energy components. However, there are limitations in the empirical understanding of scatter components inside and outside the scatter interface in this study, and Monte Carlo (MC) simulation should be performed for more accurate analysis. We assumed that the irregular patterns of the scattered radiation from the MLCs could be explained using this scatter interface. Using the MLC scatter source and scatter interface, we could appropriately account for the MLC scatter contributions at all MLC positions relative to the jaws. As shown in Fig. 8, the scatter interface shows the irregular patterns of the scattered radiation from the MLCs for jaw-defined field sizes of 10 × 10, 15 × 15, and 20 × 20 cm^2^. However, for jaw-defined field sizes of 25 × 25 and 30 × 30 cm^2^, the physical scatter interfaces were smaller than the geometric scatter interfaces. The geometric scatter interfaces for large field sizes underestimated the normalized intensity value because of an increase in the low energy component of the peripheral scattered radiation. Slightly greater maximum discrepancies between the measured and calculated *S*_c_ values were observed compared with that under smaller jaw-defined field sizes (i.e., 10 × 10, 15 × 15, and 20 × 20 cm^2^) (Fig. 8).

In this approach, line-based and area-based source functions were used to account for the complicated patterns of the scattered radiation from the MLCs. In principle, the line-based and area-based sources represent scattered radiation reaching the detector from the rounded-edge and radiation-exposed areas on the MLCs, respectively. When the MLC-defined field becomes larger at a fixed jaw setting, the scattered radiation from the MLC edge increases, whereas those from the irradiated areas of the MLCs decreases. This requires two functions, one that increases with the MLC field size and one that decreases with it, as shown in Fig. 7. The same condition is applied to the areas inside and outside the scatter interface to account for each component independently. The line-based and area-based sources change depending on the jaw-defined field size considering optimal parameters because the contribution of the scattered radiation from the jaw aperture changes with different apertures. The area-based source, expressed in Eq. (10), was based on a Gaussian function, which is widely used in obtaining good approximations of scattered radiation from radiation-exposed materials. However, for a line-based source, no such general function exists. Thus, a function was empirically chosen focusing on meeting the trend in the data rather than satisfying the physical meaning.

The parameters of the MLC scatter source model were well optimized with the objective function value below a termination tolerance of 10^−6^ using the nonlinear least squares method. Two parameters (*a* and *b*) in the line-based source and one parameter (*σ*) in the area-based source were used. Each parameter was classified based on categories 2 and 3. For the line-based source model expressed in Eq. (9), the parameter *a* helped determine the intensity of the scattered radiation from the radiation-exposed rounded-edges of the MLCs, relative to the intensity of the scattered radiation from each jaw aperture. The jaw-defined field size increased with the increase in a_out_ whereas a_in_ decreased simultaneously. It was assumed that most of the scattered radiation from the rounded-edges of the MLCs outside the scatter interface could reach the detector, while the radiation inside the scatter interface could reach the detector and the monitor chamber via backscattering. The other parameter *b* was found to decide the intensity gradient as a function of the perimeter of the MLC-defined field shape. The initial intensity gradient with a low value of *b* is high for a large jaw-defined field because the contribution of the scattered radiation from the jaw aperture increased with the increase in the jaw aperture size. The parameter *σ* in Eq. (10) for the area-based source increased exponentially with the increase in the jaw-defined field size (see Table 2). *S*_*c*,area_ was calculated using the contribution of the scattered radiation from an exposed MLC area. The high value of *σ* decreased the gradient of the cumulative distribution function for *S*_*c*,area_. The portion of the scattered radiation from an exposed MLC area, normalized with that from the reference field, decreases with the increase in the jaw-defined field size.

Figure 8 shows the variation in *S*_*c*_ with respect to the MLC-defined field sizes for five fixed jaw-defined fields. With the increase in the jaw aperture, a certain trend was more obvious: the measured *S*_*c*_ values for MLC-defined field sizes greater than a certain value were greater than the corresponding DSM-calculated *S*_*c*_ values (e.g., over 8 × 8 cm^2^ for a jaw aperture of 20 × 20 cm^2^). This is because the DSM was modeled with only jaw-defined *S*_*c*_ data, and therefore, the DEV could not consider the MLC positions in category 2 where scattered radiation from MLCs certainly exists. The scattered radiation from the MLCs was not considered in the DSM for *S*_*c*_ calculation. In this study, the MLC scatter source was developed to consider this contribution.

The effect of MLC scatter has generally been considered negligible and is not explicitly included in standard *S*_c_ calculations used for radiation therapy treatment planning. It is true that the MLC scatter contribution is insignificant in conventional treatments. However, in complicated delivery techniques, such as IMRT and VMAT, a dose to a point is a combined effect of many irregularly shaped fields including ones having leaves blocking the point in the BEV. In such cases, the relative importance of the MLC scatter contribution is significant. For example, in our clinic, a typical prostate VMAT plan having a total of 90 fields contains approximately 30 to 40 fields with the isocenter blocked. If we assume equal weighting from each field and an MLC scatter contribution of approximately 0.3%, the total MLC effect becomes approximately 0.5%, which is a justifiable level of correction considering that many correction factors (e.g., ion recombination correction factor, *P*_ion_) for output calibration have similar magnitudes. In fact, a higher MLC scatter effect is expected in more complicated plans. Five head-and-neck VMAT plans were used to verify the effectiveness of the MLC scatter source model. The VMAT plans had 178 control points for one arc. For simplicity, the measurement and calculation were performed with gantry angles fixed at 0°. The mean deviations in the central dose in the DSM and DSM + MLC cases were 3.25% and 1.02%, respectively, for the five plans. This shows that the monitor unit (MU) calculation accuracy for IMRT or VMAT plans was improved when the proposed method was incorporated in the MU calculation program. As future work, comprehensive analysis including various tumor sites and types of plans will be conducted to demonstrate the advantage of this method.

In this work, we developed an MLC scatter source and a scatter interface to account for the scattered radiation from various MLC-defined fields. It was difficult to simplify the model because the behavior of radiation scattered from the complicated structure of MLC leaves is not simple. MC simulations that can track the life of each simulated photon with all interaction events in surrounding materials will be performed as a future work. With this, we can analyze the accurate energy fluence within the scatter interfaces to define all the MLC scatter components more clearly and further improve the model.

## Conclusion

An extra-focal source model, namely the MLC scatter source, was developed to accurately calculate the scatter components from the head of a linear accelerator. The MLC scatter source comprises line-based and area-based sources. We demonstrated that in conjunction with the conventional DSM, the developed MLC scatter source could improve the accuracy of *S*_*c*_ calculation for both square and irregular fields.
